# Thrombocytopenia as a prognostic marker in COVID-19 patients: diagnostic test accuracy meta-analysis

**DOI:** 10.1017/S0950268821000236

**Published:** 2021-01-29

**Authors:** Raymond Pranata, Michael Anthonius Lim, Emir Yonas, Ian Huang, Sally Aman Nasution, Siti Setiati, Idrus Alwi, Raden Ayu Tuty Kuswardhani

**Affiliations:** 1 Faculty of Medicine, Universitas Pelita Harapan, Tangerang, Indonesia; 2 Faculty of Medicine, Universitas YARSI, Jakarta, Indonesia; 3 Department of Internal Medicine, Faculty of Medicine, Universitas Padjadjaran, Hasan Sadikin General Hospital, Bandung, Indonesia; 4 Division of Cardiology, Department of Internal Medicine, Faculty of Medicine, Universitas Indonesia/Cipto Mangunkusumo National General Hospital, Jakarta, Indonesia; 5 Division of Geriatrics, Department of Internal Medicine, Faculty of Medicine, Faculty of Medicine, Universitas Indonesia-Cipto Mangunkusumo General Hospital, Jakarta; 6 Division of Geriatrics, Department of Internal Medicine, Faculty of Medicine, Udayana University, Sanglah Teaching Hospital, Denpasar, Bali, Indonesia

**Keywords:** Coronavirus, mortality, platelet, severe, thrombocyte

## Abstract

This systematic review and meta-analysis aimed to evaluate thrombocytopenia as a prognostic biomarker in patients with coronavirus disease 2019 (COVID-19). We performed a systematic literature search using PubMed, Embase and EuropePMC. The main outcome was composite poor outcome, a composite of mortality, severity, need for intensive care unit care and invasive mechanical ventilation. There were 8963 patients from 23 studies. Thrombocytopenia occurred in 18% of the patients. Male gender (*P* = 0.037) significantly reduce the incidence. Thrombocytopenia was associated with composite poor outcome (RR 1.90 (1.43–2.52), *P* < 0.001; *I*^2^: 92.3%). Subgroup analysis showed that thrombocytopenia was associated with mortality (RR 2.34 (1.23–4.45), *P* < 0.001; *I*^2^: 96.8%) and severity (RR 1.61 (1.33–1.96), *P* < 0.001; *I*^2^: 62.4%). Subgroup analysis for cut-off <100 × 10^9^/l showed RR of 1.93 (1.37–2.72), *P* < 0.001; *I*^2^: 83.2%). Thrombocytopenia had a sensitivity of 0.26 (0.18–0.36), specificity of 0.89 (0.84–0.92), positive likelihood ratio of 2.3 (1.6–3.2), negative likelihood ratio of 0.83 (0.75–0.93), diagnostic odds ratio of 3 (2, 4) and area under curve of 0.70 (0.66–0.74) for composite poor outcome. Meta-regression analysis showed that the association between thrombocytopenia and poor outcome did not vary significantly with age, male, lymphocyte, d-dimer, hypertension, diabetes and CKD. Fagan's nomogram showed that the posterior probability of poor outcome was 50% in patients with thrombocytopenia, and 26% in those without thrombocytopenia. The Deek's funnel plot was relatively symmetrical and the quantitative asymmetry test was non-significant (*P* = 0.14). This study indicates that thrombocytopenia was associated with poor outcome in patients with COVID-19.

PROSPERO ID: CRD42020213974

## Introduction

The coronavirus disease 2019 (COVID-19) remains a significant concern in many countries throughout the world. The number of new cases continues to grow worldwide, and this situation expands the capacity of healthcare resources to its limit. A decisive mitigation approach and effective allocation of resources are essential in slowing the progress of the pandemic, therefore, early assessments to forecast the clinical course of the disease are critical. The levels of several inflammatory parameters are often elevated in the context of severe COVID-19, given the excessive immune response to viral infection resulting in a hyperinflammatory state [1]. Basic investigation such as complete blood count may prove useful in predicting the prognosis of COVID-19 patients. In addition to a reduced lymphocyte count [2], the platelet count can be found in low levels in individuals with severe COVID-19 infection. This systematic review and meta-analysis aimed to evaluate thrombocytopenia as a prognostic biomarker in patients with COVID-19. We performed a diagnostic test accuracy meta-analysis to evaluate whether thrombocytopenia can be a suitable prognostic biomarker.

## Material and methods

This is a Preferred Reporting Items for Systematic Reviews and Meta-Analyses (PRISMA) compliant systematic review and meta-analysis. The protocol was registered in International Prospective Register of Systematic Review (PROSPERO) with ID: CRD42020213974.

### Eligibility criteria

In this study we include research articles (both prospective and retrospective observational studies) and letters containing research data that report COVID-19 confirmed adults with information on thrombocytopenia (categorical) and mortality/severity/intensive care unit (ICU) care/invasive mechanical ventilation (IMV) (categorical).

We excluded preprints, abstract-only articles, reviews, editorials/commentaries, non-research letters, studies with less than 20 patients, case reports and non-English language articles. Studies reporting only continuous variables of platelets were excluded. Preprint articles were excluded because of the varying credibility [3].

### Search strategy and study selection

We performed a systematic literature search using PubMed, Embase and EuropePMC with keywords ‘COVID-19’ OR ‘SARS-CoV-2’ OR ‘2019-nCoV’ AND ‘thrombocytopenia’ OR ‘low platelet’ AND ‘Mortality’ OR ‘Death’ OR ‘non-survivor’ OR ‘severity’ OR ‘disease progression’ or ‘intubation’ OR ‘mechanical ventilation’ OR ‘intensive care unit’ from inception up until 12 October 2020. The PubMed (MEDLINE) search strategy was ((COVID-19) OR (SARS-CoV-2) OR (2019-nCoV)) AND ((thrombocytopenia) OR (low platelet)) AND ((mortality) OR (death) OR (non-survivor) OR (severity) OR (disease progression) or (intubation) OR (mechanical ventilation) OR (intensive care unit)). After recording the initial search and removal of duplicates, two independent authors performed title/abstract screening on the basis of inclusion and exclusion criteria.

### Data extraction

Two authors independently performed data extraction of the included studies with the help of standardised extraction forms containing the rows and columns related to the first author, year of publication, study design, age, gender, hypertension, diabetes, chronic kidney diseases (CKD), lymphocyte counts, d-dimer levels and outcome of interests.

The main outcome was composite poor outcome, which is a composite of mortality, severity, ICU care and IMV. Mortality was defined as clinically validated mortality/death/non-survivor.

Severity was defined according to the included studies' parameters, which might be WHO−China Joint Mission on coronavirus disease definition, disease progression or others. ICU care and mechanical ventilation were defined as the need for ICU admission and IMV/intubation, respectively. The pooled effect estimates were presented as risk ratios (RRs). Additionally, diagnostic meta-analysis will use sensitivity, specificity, positive likelihood ratio (PLR), negative likelihood ratio (NLR), diagnostic odds ratio (DOR) and area under curve (AUC) for the effect estimates.

### Risk of bias assessment

Risk of bias assessment of individual studies was performed by two independent authors using the Newcastle−Ottawa Scale [4], and any discrepancies were resolved by discussion.

### Statistical analysis

To perform statistical analysis, we used STATA 16 (Stata Corp, College Station, TX, USA). We performed the meta-analysis of proportion for patients with thrombocytopenia. To perform pooled analysis for RRs, we used the DerSimonian & Laird method and Mantel−Haenszel with random-effects model regardless of heterogeneity. *P*-values were considered as statistically significant if ≤0.05. Heterogeneity of the pooled estimate was assessed using I-squared (*I*^2^) and Cochrane *Q* test, a value of >50% or *P*-value <0.10 indicates a statistically significant heterogeneity. Restricted-maximum likelihood random-effects meta-regression was performed using one covariate at a time for age, gender, hypertension, diabetes mellitus, lymphocyte and d-dimer [5]. For the diagnostic meta-analysis, we displayed forest plot of sensitivity and specificity (a bivariate model) along with a summary receiver operating characteristic (SROC) curve [6]. Fagan's nomogram was plotted to evaluate relationship between the prior probability and posterior test probability [7]. Deek's funnel plot and asymmetry test were used to evaluate publication bias. We performed univariable meta-regression and subgroup analyses for the output of diagnostic meta-analysis using age, male, lymphocytes, d-dimer, hypertension, diabetes and CKD as covariates.

## Results

**Fig. 1.**
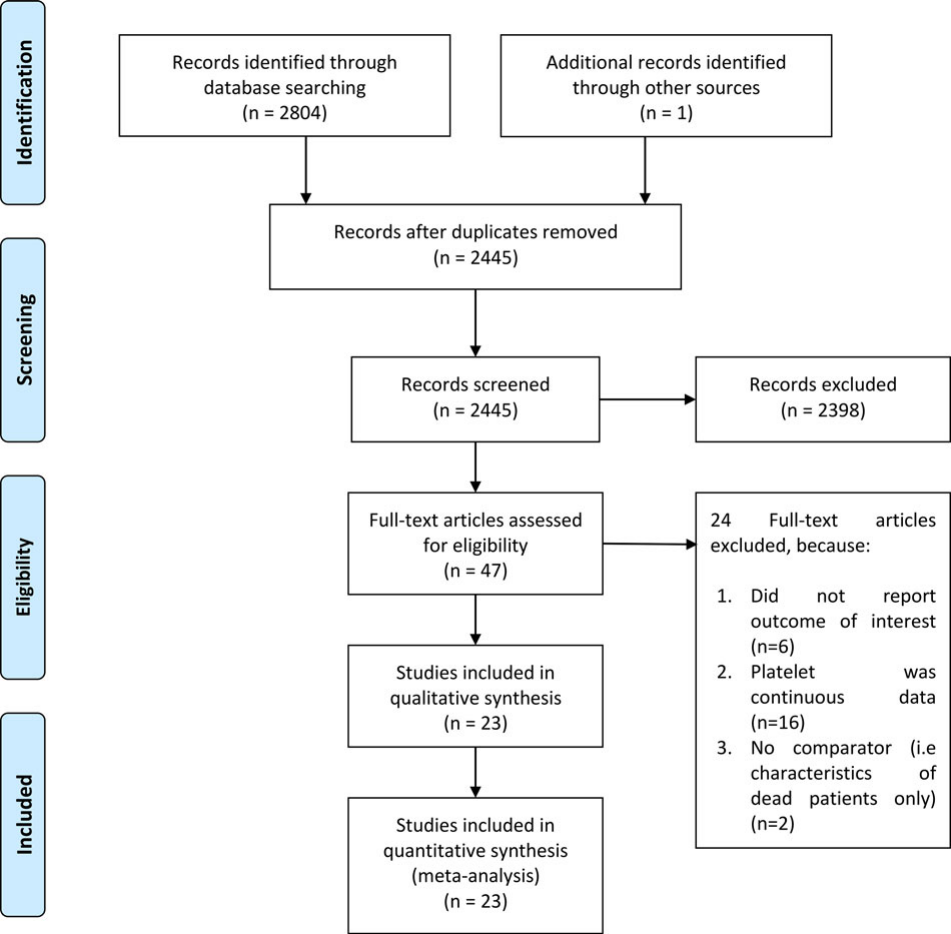
PRISMA flowchart.

There were 8963 patients from 23 eligible studies included in the systematic review and meta-analysis (Fig. 1) [8–30]. The baseline characteristics of the included studies can be seen in Table 1. Thrombocytopenia occurred in 18% (13–23%) of the patients. Male gender (coefficient: −0.004, *P* = 0.034), but not age (*P* = 0.290), lymphocyte (*P* = 0.905), d-dimer (*P* = 0.195), hypertension (*P* = 0.355), diabetes (*P* = 0.450) and CKD (*P* = 0.658) significantly influence the incidence. The incidence of composite poor outcome was 30%.

**Table 1.** Baseline characteristics of the included studies

References	Study design	Sample size (*n*)	Mean age (years)	Male (%)	HT (%)	DM (%)	CKD (%)	Lymphocytes (10^9^)	D-dimer (mcg/ml)	Outcome	Cut-off for Thrombocytopenia (*10^9^/l)	NOS
[8]	PO	373	52.78	67	–	–	–	–	–	Mortality	<150	7
[9]	PO	178	64	59.6	32.6	17.4	1.7	1.2	0.56	Severity	<100	7
[20]	RO	21	56	81	23.8	14.3	–	0.9	0.5	Severity	<100	7
[24]	RO	44	>65 (56.8)	36.3	34.1	15.9	–	–	–	Severity	<150	7
[25]	RO	393	62.2	60.6	50.1	25.2	–	–	Dimer >0.5 (36.4)	IMV	<150	6
[26]	RO	1099	47	59.1	15	7.4	0.7	1	Dimer >0.5 (46.4)	Severity	<150	7
[27]	RO	98	55.4	38.8	30.6	9.2	–	1.4	–	ICU Care	?	7
[28]	RO	41	49	73	15	20	–	0.8	0.5	ICU Care	<100	6
[29]	RO	110	56.9	43.6	33.6	26.4	–	1.4	4.4 Dimer >0.5 (76.3)	Severity	<140	9
[30]	RO	59	64	49	42	15	–	1.1	6.1	ICU Care	<100	7
[10]	RO	63	48	85	32	32	6.4	1.1	0.6 Dimer >0.55 (59)	ICU Care	<125	6
[23]	RO	611	64	54	30	16		1	1.24	Severity	<100	7
[11]	RO	548	60	50.9	30.3	15.1	1.8	-	–	Severity	<150	9
[12]	RO	1190	57	53.4	26.1	12.2	2.6	1.2	0.9	Mortality	<100	9
[13]	RO	756	69	62.2	37.8	28.6	13.5		Dimer >0.50 (87.8)	Mortality	<130	9
[14]	RO	113	53.8	62.8	19.5	14.2	5.3	1.2	–	Mortality	<150	7
[15]	RO	135	47	53.3	9.6	8.9	–	1.1	0.4	Severity	<125	7
[16]	RO	276	51	56.2	17	5.1	–	1.1	Dimer >0.50 (53.7)	Severity	<150	7
[17]	RO	239	62.5	59.8	43.9	18.4	–	0.6	–	Mortality	<125	9
[18]	RO	1476	58.7	52.3	–	–	–	–	–	Mortality	<125	8
[19]	RO	788	45.8	51.6	16	7.2	0.9	1.2	–	Severity	<100	9
[21]	RO	161	45	49.7	13.7	4.3		1.1	–	Severity	<100	7
[22]	RO	191	56	62	30	19	1	1	0.8 Dimer >0.50 (68)	Mortality	<100	7

CKD, chronic kidney disease; DM, diabetes mellitus; HT, hypertension; ICU, intensive care unit; IMV, invasive mechanical ventilation; PO, prospective observational; RO, retrospective observational; NOS, Newcastle−Ottawa scale.

### Thrombocytopenia and composite outcome

**Fig. 2.**
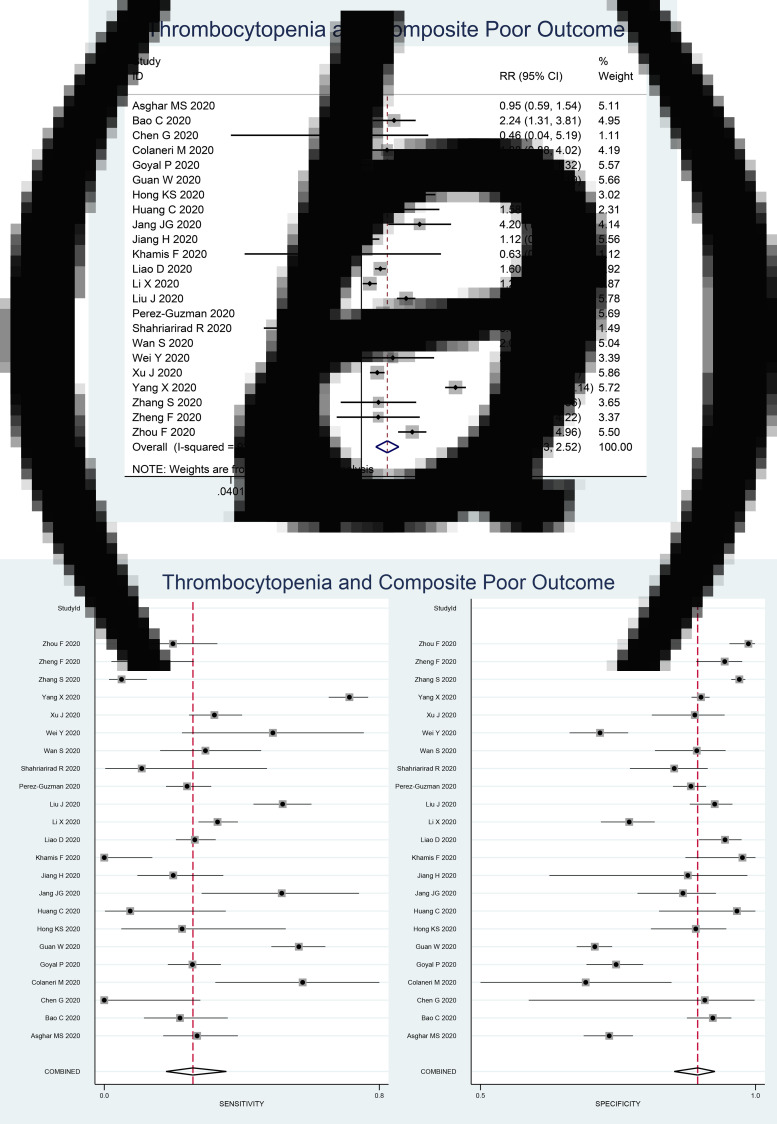
Forest-plot for thrombocytopenia and composite poor outcome (a) and pooled sensitivity and specificity (b).

Thrombocytopenia was associated with composite poor outcome (RR 1.90 (1.43–2.52), *P* < 0.001; *I*^2^: 92.3%, *P* < 0.001) (Fig. 2a). Subgroup analysis showed that thrombocytopenia was associated with mortality (RR 2.34 (1.23–4.45), *P* < 0.001; *I*^2^: 96.8%, *P* < 0.001) and severity (RR 1.61 (1.33–1.96), *P* < 0.001; *I*^2^: 62.4%, *P* < 0.001). Subgroup analysis for cut-off <100 × 10^9^/l (9 studies) showed RR of 1.93 (1.37–2.72), *P* < 0.001; *I*^2^: 83.2%, *P* < 0.001). Subgroup analysis for cut-off <150 × 10^9^/l (seven studies) was borderline significant (RR 1.39 (0.99–1.95), *P* = 0.058; *I*^2^: 76.2%, *P* < 0.001).

Meta-regression analysis showed that the association did not vary significantly with age (*P* = 0.534), male (*P* = 0.117), lymphocyte (*P* = 0.910), d-dimer (*P* = 0.751), hypertension (*P* = 0.0647), diabetes (*P* = 0.812) and CKD (*P* = 0.509).

### Diagnostic meta-analysis

Thrombocytopenia had a sensitivity of 0.26 (0.18–0.36), specificity of 0.89 (0.84–0.92), PLR of 2.3 (1.6–3.2), NLR of 0.83 (0.75–0.93), DOR of 3 (2, 4) and AUC of 0.70 (0.66–0.74). Pooled sensitivity and specificity is displayed in Figure 2b.

**Fig. 3.**
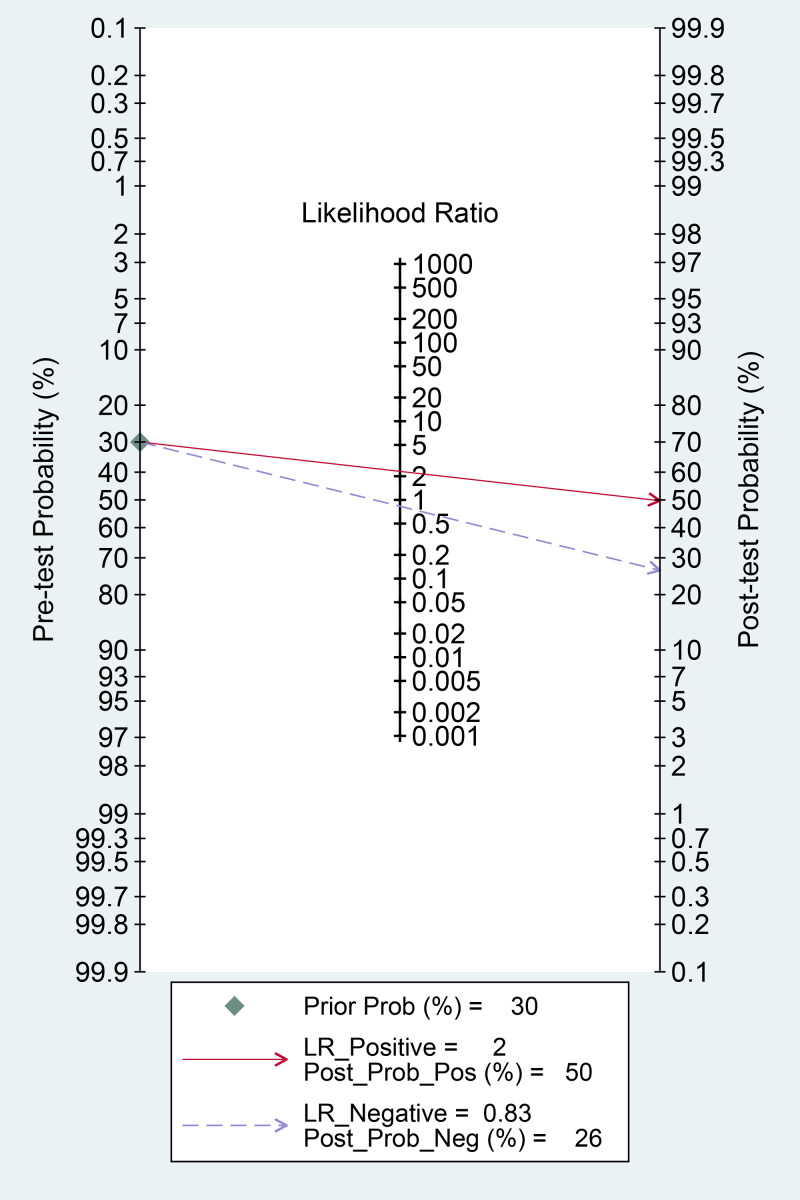
Fagan's nomogram.

**Fig. 4.**
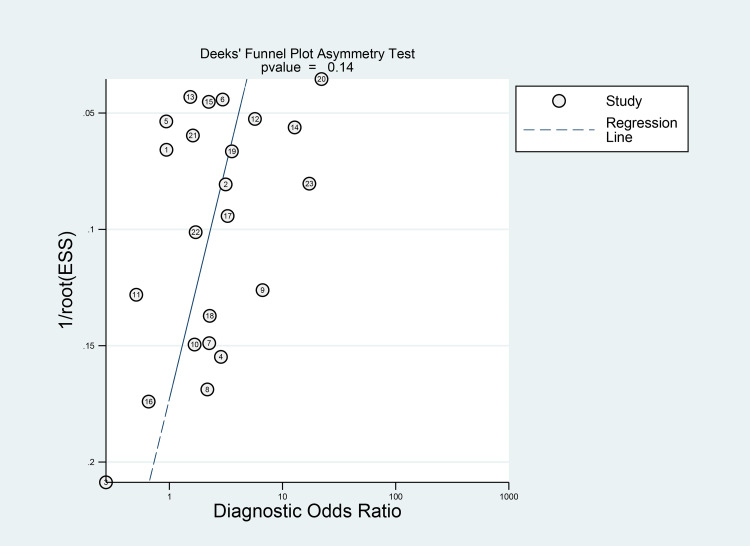
Deek's funnel plot and asymmetry test. The vertical axis displays the inverse of the square root of the effective sample size (1/root(ESS)).

Fagan's nomogram showed that the posterior probability of poor outcome was 50% in patients with thrombocytopenia, and 26% in those without thrombocytopenia (Fig. 3). The Deek's funnel plot was relatively symmetrical with respect to the regression line and the asymmetry test was non-significant (*P* = 0.14) (Fig. 4).

**Fig. 5.**
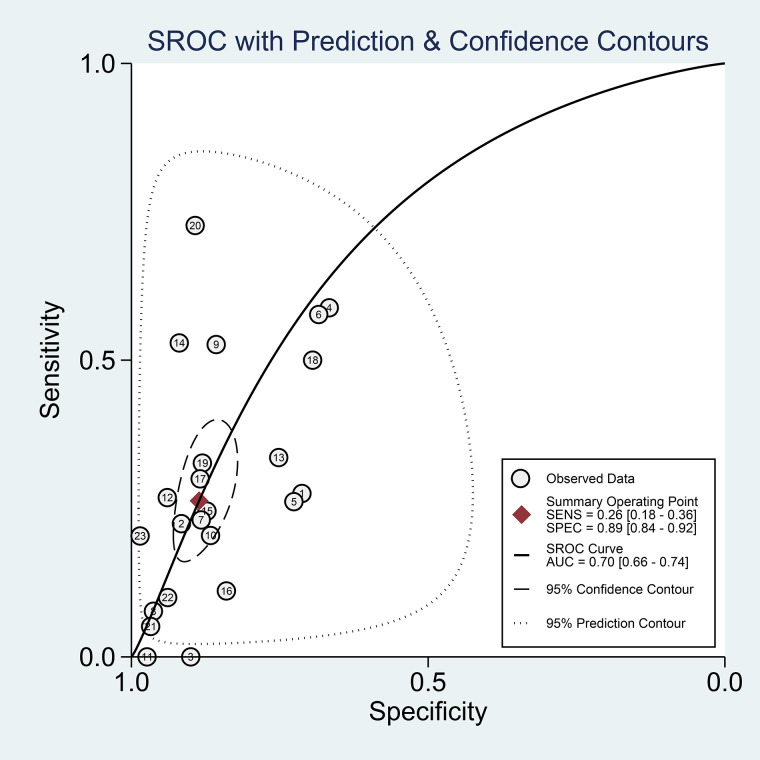
Summary receiver operating characteristic (SROC) with prediction and confidence contours.

**Fig. 6.**
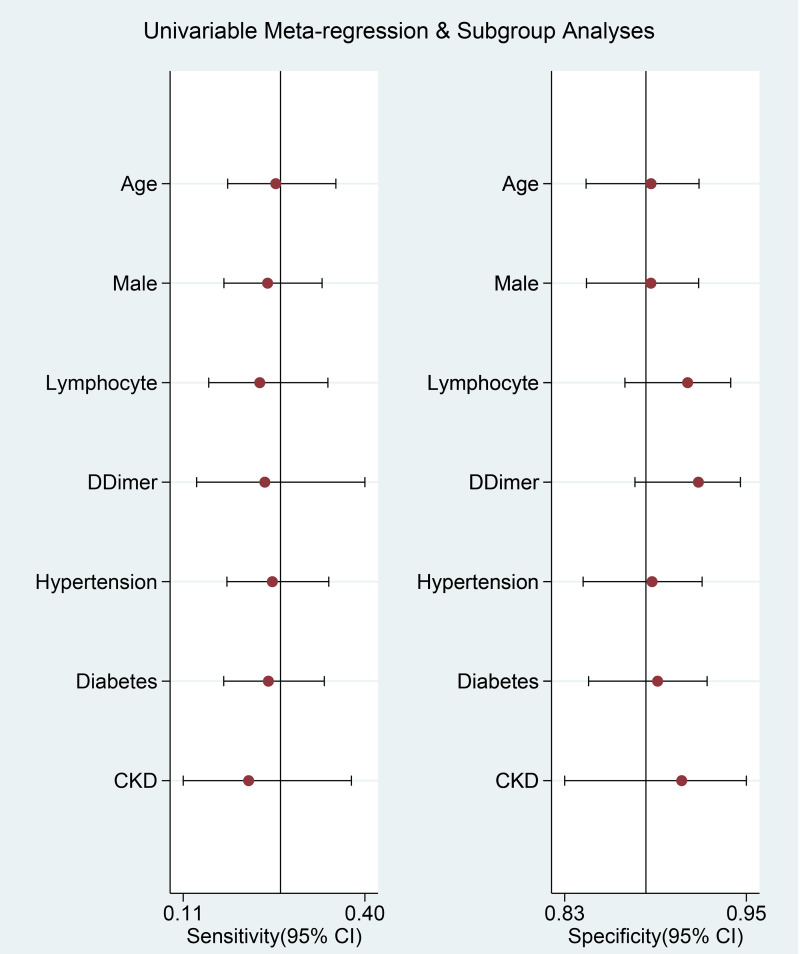
Univariable meta-regression and subgroup analyses. Vertical lines represent pooled sensitivity and specificity, respectively.

A curve for prediction of composite poor outcome was generated (Fig. 5). The 95% prediction region was wide, however, meta-regression analysis showed that no reasons for the heterogeneity in the selected covariates, age, male, lymphocytes, d-dimer, hypertension, diabetes and CKD. Univariable meta-regression and subgroup analyses are displayed in Figure 6.

## Discussion

This study indicates that thrombocytopenia was associated with poor outcome in patients with COVID-19. Diagnostic test accuracy indicates sensitivity of 26% and specificity of 89% with AUC of 0.70. Meta-regression analysis showed that the association or diagnostic accuracy was not significantly influenced by age, male, lymphocytes, d-dimer level, hypertension, diabetes and CKD. There are varying cut-off points for thrombocytopenia, a cut-off point of <100 × 10^9^/l showed a stronger association compared to <150 × 10^9^/l. The varying cut-off point might also cause heterogeneity. Meta-regression analysis also showed that male gender was associated with reduced incidence of thrombocytopenia.

Comorbidities, such as diabetes mellitus, hypertension, chronic lung disease, cardiac disease and CKD [31–36] as well as individuals with advanced age and obesity are at higher risk for severe COVID-19 and mortality [37, 38]. Several parameters such as C-reactive protein (CRP), procalcitonin (PCT), ferritin and D-dimer are frequently elevated in patients with poor prognosis [1]. Based on the aforementioned possible factors, meta-regression analyses were performed for several variables that may affect the prediction derived from thrombocytopenia, these include age, male, lymphocytes, d-dimer, hypertension, diabetes and CKD. On admission, a complete blood count is almost always ordered and often repeated at the discretion of the treating doctor, thus, did not add to the treatment costs. Since platelet count is simple, easy and inexpensive to perform, it is an ideal clinical prognostication tools. Low platelet count is a common laboratory finding in patients with severe COVID-19, in our meta-analysis, we found that thrombocytopenia was present in 18% of the patients [9, 18].

Coagulopathy is one of the serious complications that can develop in patients with COVID-19, as well as acute respiratory distress syndrome (ARDS), cardiopulmonary collapse and multi-organ failure (MOF) [39]. Alteration in bleeding and clotting parameters can contribute to the development of coagulopathy-related events, such as pulmonary emboli (PE) and disseminated intravascular coagulation (DIC). In some patients, the prothrombin time (PT) and activated partial thromboplastin time (aPTT) is prolonged, while others may have a PT [40, 41]. Haematological changes, including lymphopenia and thrombocytopenia but normal white blood cell count, are also commonly observed in individuals contracted to severe acute respiratory syndrome coronavirus 2 (SARS-CoV-2) [2]. However, it is still unclear how the novel coronavirus influence the haematopoietic system.

The pathophysiology of thrombocytopenia in COVID-19 is hypothetically caused by the alteration of platelets' production and consumption (and/or destruction) [42]. It affects platelets' production by either directly or indirectly affecting the haematopoietic stem cells (HSCs), reduction of thrombopoietin production, and megakaryocyte maturation due to increase of specific inflammatory cytokines [43], and decrease of supplementary haematopoietic progenitor in the pulmonary vessels due to COVID-19-induced lung damage [44]. Moreover, extensive lung damage and disseminated intravascular coagulation due to hyperinflammation cause further thrombocytopenia in COVID-19 due to the increase of platelet consumption [42].

Coronaviruses may directly infect haematopoietic and bone marrow cells and cause aberrant haematopoiesis. Human aminopeptidase N (CD13) is a metalloprotease found on lung epithelial cells' surface, smooth muscle cells, fibroblasts and epithelial cells in the kidneys and small intestine, may serve as a receptor for the entry of novel coronavirus. It is speculated that the virus may invade bone marrow cells and platelets through CD13 receptors and cause growth inhibition and apoptosis, resulting in haematopoiesis dysfunction, reduced primary platelet production and eventual thrombocytopenia [18, 40, 45].

Cytokine storm, a condition in which human body releases large amounts of inflammatory cytokines and cause hyperinflammation, is thought to play a central role in the pathophysiology of COVID-19 [1, 39]. Excessive activation and proliferation of mononuclear macrophage system leads to secondary haemophagocytic lymphohistiocytosis (sHLH), in which cytokines are released excessively and an enormous number of blood cells are engulfed, contributing to reduced peripheral blood platelets. This proinflammatory state also causes immune damage to various organs, including the lungs, and is thought to affect the haematopoietic progenitor cells in the bone marrow, leading to reduced platelet synthesis [40, 41].

The lung is a reservoir for haematopoietic progenitors and a site of platelet biogenesis. Lung injury in COVID-19 pneumonia may be exacerbated by persistent hypertension and oxygen toxicity, leading to consolidation changes and pulmonary fibrosis. It is found that a considerable number of megakaryocytes releases platelet dynamically into pulmonary circulation [44]. Injured capillary beds and diffuse alveolar damage in the lungs cause trapping of megakaryocyte and inhibit platelets' release from megakaryocytes, which causes reduced platelet formation in the systemic circulation [18, 45]. Also, injured pulmonary endothelial cells and lung tissues will activate the coagulation system, resulting in platelet aggregation and micro-thrombosis in the lungs, which increases platelet consumption [40].

Elevated levels of autoantibodies and immune complexes in patients with SARS-CoV-2 infection may result in platelet destruction and clearance by the immune system. The exact pathogenesis of immune-mediated thrombocytopenia in COVID-19 is still unclear, however, reticuloendothelial cells may recognise platelets as target tissue given the presence of antibodies and immune complexes deposited on the platelet surface. Furthermore, antibodies generated when the virus invades human cells may specifically bind to platelet antigens via molecular mimicry, causing substantial damage to the circulating platelets [40, 45, 46]. The reason why thrombocytopenia is independently associated with poor outcome is biologically plausible because it might reflect a greater viral load of SARS-CoV-2, higher inflammatory response and extensive lung damage, as we previously described the hypothetical mechanism behind this phenomenon.

Sociodemographic may influence the findings in this meta-analysis, for example, certain infections such as dengue virus may cause thrombocytopenia [47]. Thrombocytopenia caused by dengue virus may differ from those caused by sepsis and disseminated intravascular coagulation, or COVID-19 [40, 48, 49]. Concurrent infections with such diseases were not reported among the studies. The prevalence of these infections may be higher in tropical regions [50, 51]. Clinical applications should not be generalised without considering these factors.

### Clinical implications

This meta-analysis indicate that thrombocytopenia was associated with increased composite poor outcome with RR of 1.90. It has a low sensitivity but high specificity, thus is best used to rule in rather to rule out the possibility of composite poor outcome. The sensitivity and specificity are highly heterogeneous as demonstrated by Figure 2. In a 30% pre-test probability for composite poor outcome, thrombocytopenia may increase the probability to 50%. Meanwhile, Deek's funnel plot asymmetry test indicate low risk of publication bias. SROC curve showed a AUC of 0.70, indicating an acceptable diagnostic performance. Meta-regression analysis indicate that several assigned comorbidities did not affect the sensitivity and specificity for thrombocytopenia to predict poor outcome. Thus, thrombocytopenia might be used to rule in composite poor outcome, but not to rule it out. Finding from subgroup analysis based on cut-off points may indicate that a lower cut-off point was associated with a higher risk. To enhance the sensitivity and specificity, it is better to construct a prediction model using several variables.

### Limitations

Most of the studies did not report the number of possible co-infections that can potentially cause thrombocytopenia or the number of bacterial infections. The definition for thrombocytopenia still varies among the included studies. Drug therapy, such as anticoagulants use were rarely reported by the studies.

## Conclusion

This study indicates that thrombocytopenia was associated with poor prognosis in patients with COVID-19. Diagnostic test accuracy indicates sensitivity of 26% and specificity of 89% with AUC of 0.70.

## Data Availability

All data generated or analysed during this study are included in this published article
